# Severe neurodevelopmental disease caused by a homozygous *TLK2* variant

**DOI:** 10.1038/s41431-019-0519-x

**Published:** 2019-09-26

**Authors:** Ana Töpf, Yavuz Oktay, Sunitha Balaraju, Elmasnur Yilmaz, Ece Sonmezler, Uluc Yis, Steven Laurie, Rachel Thompson, Andreas Roos, Daniel G. MacArthur, Ahmet Yaramis, Serdal Güngör, Hanns Lochmüller, Semra Hiz, Rita Horvath

**Affiliations:** 1 John Walton Muscular Dystrophy Research Centre, Institute of Genetic Medicine, Newcastle University, Newcastle upon Tyne, UK; 2 Izmir Biomedicine and Genome Center, Dokuz Eylul University Health Campus, Izmir, Turkey; 3 Department of Medical Biology, School of Medicine, Dokuz Eylul University, Izmir, Turkey; 4 Izmir International Biomedicine and Genome Institute, Dokuz Eylul University, Izmir, Turkey; 5 Department of Paediatric Neurology, School of Medicine, Dokuz Eylul University, Izmir, Turkey; 6 CNAG-CRG, Centre for Genomic Regulation, Barcelona Institute of Science and Technology, Barcelona, Spain; 7 Leibniz Institut für Analytische Wissenschaften, ISAS, Dortmund, Germany; 8 Pediatric Neurology, University Children’s Hospital, University of Duisburg-Essen, Faculty of Medicine, Essen, Germany; 9 Analytic and Translational Genetics Unit, Massachusetts General Hospital, Boston, MA USA; 10 Program in Medical and Population Genetics, Broad Institute of MIT and Harvard, Cambridge, MA USA; 11 Pediatric Neurology Clinic, Diyarbakir Memorial Hospital, Diyarbakir, Turkey; 12 Department of Paediatric Neurology, Faculty of Medicine, Turgut Ozal Research Center, Inonu University, Malatya, Turkey; 13 Department of Neuropediatrics and Muscle Disorders, Faculty of Medicine, Medical Center—University of Freiburg, Freiburg, Germany; 14 Children’s Hospital of Eastern Ontario Research Institute, University of Ottawa, Ottawa, ON Canada; 15 Division of Neurology, Department of Medicine, The Ottawa Hospital, Ottawa, ON Canada; 16 Department of Clinical Neurosciences, University of Cambridge School of Clinical Medicine, Cambridge Biomedical Campus, Cambridge, UK

## Abstract

A distinct neurodevelopmental phenotype characterised mainly by mild motor and language delay and facial dysmorphism, caused by heterozygous de novo or dominant variants in the *TLK2* gene has recently been described. All cases reported carried either truncating variants located throughout the gene, or missense changes principally located at the C-terminal end of the protein mostly resulting in haploinsufficiency of *TLK2*. Through whole exome sequencing, we identified a homozygous missense variant in *TLK2* in a patient showing more severe symptoms than those previously described, including cerebellar vermis hypoplasia and West syndrome. Both parents are heterozygous for the variant and clinically unaffected highlighting that recessive variants in *TLK2* can also be disease causing and may act through a different pathomechanism.

## Introduction

The *TLK2* gene encodes the tousled-like kinase 2, a nuclear serine/threonine kinase known to be involved in DNA replication and chromatin assembly by phosphorylating chromatin assembly factors, such as ASF1 and regulating histone usage [1]. *TLK2* was suggested as a candidate gene for intellectual disability (ID) in a meta-analysis of de novo variants in 2104 exome trios [2], and recently Reijnders et al. [3] associated it with ID within a broader phenotype in a cohort of 40 individuals from 38 families. The cases presented with a distinct neurodevelopmental phenotype characterised by mild motor and language delay, behavioural problems, facial dysmorphism and gastro-intestinal symptoms caused by heterozygous de novo or dominant variants in *TLK2*. Truncating (i.e. nonsense, frameshift and splice site) variants located throughout the gene, or missense changes principally located at the C-terminal end of the protein were identified amongst the cohort. RNA analysis from patient derived cell lines suggested that the variants resulted in loss-of-function and that the most likely disease mechanism was haploinsufficiency of *TLK2*. Here, we report a child carrying a homozygous missense variant in *TLK2* showing more severe symptoms than those previously described.

## Subject and methods

Patient and family members were recruited at the Department of Paediatric Neurology, Izmir, Turkey. Written informed consent was obtained. Whole exome sequencing (WES) was performed by the Genomics Platform at the Broad Institute of MIT and Harvard, Cambridge, USA. Libraries were created with an Illumina exome capture (38 Mb target) and sequenced with a mean target coverage of >80 × . Exome sequencing data were processed and analysed on the RD-Connect Genome-Phenome Analysis Platform (https://platform.rd-connect.eu/genomics). Likely pathogenic variants, affecting the function of the gene, and potentially causing disease, were identified applying standard filtering criteria: minor allele frequency <1%, and high to moderate variant effect predictor (i.e. nonsense, splice site, frameshift, in-frame and non-synonymous variants). Shortlisted variants were interrogated for their predicted in silico deleteriousness and previous known association with human disease. Genomic and phenotypic data have been submitted to RD-Connect under accession numbers E287350, E180542 and E227711 (index case, mother and father, respectively) where they can be accessed under a controlled access agreement (https://platform.rd-connect.eu/datasubmission/). Annotation of the *TLK2* gene is according to NM_006852.3.

## Clinical description

**Fig. 1:**
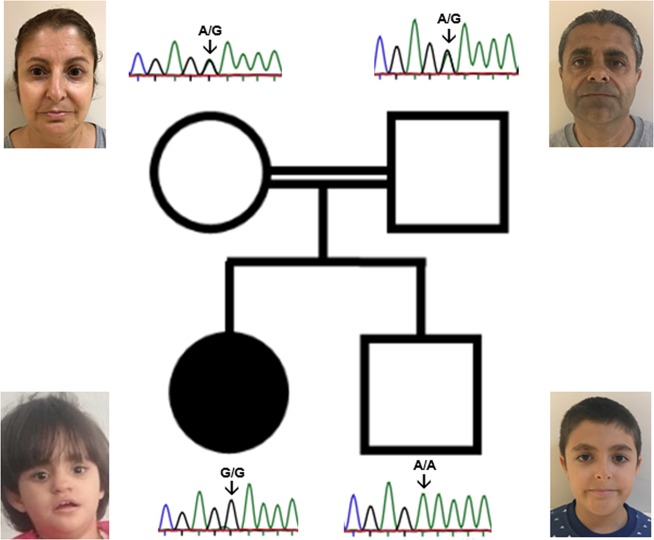
Pedigree and Sanger segregation of the *TLK2* variant (hg19 chr17:60599574 A>G). The index case is homozygous for the alternate allele (G/G), whereas her unaffected brother is homozygous wild type (A/A) and his parents are heterozygous (A/G) (indicated by an arrow). No dysmorphic features are observed in the healthy family members, including those who are carriers for the *TLK2* variant

**Fig. 2:**
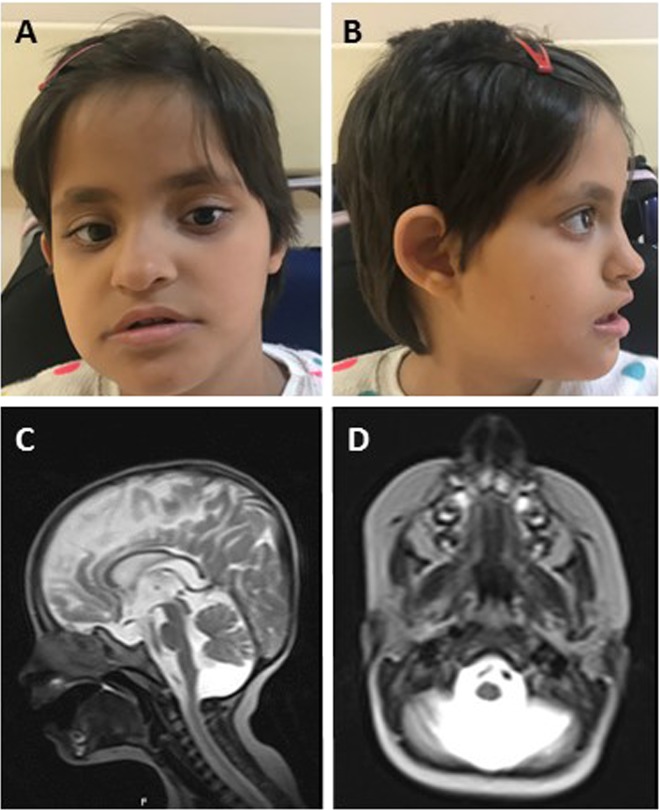
Clinical findings. **a**, **b** Patient photographs at 6 years of age showing facial dysmorphism: telecanthus and upslanting palpebral fissures, thin vermilion upper lip, big mouth and broad nasal bridge; **b** Sagittal T2-weighted cranial MR images showing cerebellar vermis hypoplasia (Dandy-Walker variant); **c** Axial T2-weighted cranial MR images showing cystic dilatation of the cerebellum indicating cerebellar vermis hypoplasia

The patient is a 6-year-old girl and the second child of unaffected consanguineous parents of Turkish origin; her 9-year-old brother is healthy (Fig. 1). She was born at term but had intrauterine growth retardation (birth weight 2400 g). Intrauterine foetal movements were reduced and she had hypotonia and hip dislocation at birth. She presented with epileptic spasms at the age of 6 months. Her EEG showed hypsarrhythmia suggesting West syndrome. She has been seizure-free since 3 years of age. On clinical examination at 6 years of age her psychomotor and speech development were severely delayed, she could sit without support but was unable to walk or speak. She had microcephaly, coarse facial appearance and dysmorphic facial features including telecanthus, upslanting palpebral fissures, thin vermilion upper lip, big mouth and broad nasal bridge (Fig. 2a, b), and a hemangioma on the left parietal scalp. In addition, she had feeding difficulties, dysphagia, peripapillary atrophy on fundoscopic examination and conductive hearing loss in the left ear. Neurological examination detected spastic tetraparesis with increased deep tendon reflexes, head titubation, trunk ataxia and dysmetria. She also presented with constipation and behavioural problems, including aggression and irritability. Her brain MRI at age 2 years showed cerebellar vermis hypoplasia (Dandy-Walker variant) and dilatation of lateral ventricles (Fig. 2c, d). Abdominal MRI detected medullary nephrocalcinosis and hydronephrosis.

## Results

Trio analysis of the patient and her parents identified a homozygous missense variant in the *TLK2* gene (hg19 chr17: g.60599574 A>G; c.163 A>G; p.(Lys55Glu)), which affects a highly conserved amino acid. The variant is predicted to be damaging by all tested in silico tools, is amongst the top 0.2% most deleterious variants (CADD score 27) [4] and is classified as ‘likely pathogenic’ by the ACMG Guidelines [5]. This variant has been detected only once, in a heterozygous European, out of 122,547 individuals in gnomAD and is absent from a cohort of 1182 ethnically-matched Turkish control individuals (TUBITAK MAM-GMBE dataset; http://gmbe.mam.tubitak.gov.tr/en). Sanger sequencing showed that the variant is not present in her healthy brother, and that both parents are heterozygous carriers. On examination they were clinically unaffected and showed no signs of facial dysmorphism or cognitive impairment (Fig. 1).

## Discussion

We report a homozygous missense variant in *TLK2* in a patient with a neurodevelopmental disorder with severe motor and language delay, West syndrome, pontocerebellar hypoplasia, behavioural problems, facial dysmorphism and gastro-intestinal symptoms. The clinical presentation fits what has been described by Reijnders et al. [3] for heterozygous *TLK2* patients and the dysmorphic features were remarkably similar. Our index case, however, presented with more severe symptoms, with profound ID, spastic tetraparesis and a structural brain anomaly. Whilst conductive hearing loss, microcephaly and non-specific brain anomalies were present in a minority of reported cases (<25%), we are not aware of cerebellar vermis hypoplasia, which we observed in the homozygous individual. Although the majority of the patients reported by Reijnders had language and cognitive delay (92% and 74%, respectively), this was mainly mild ID (IQ 50–70). In contrast, our patient presents severe neurodevelopmental delay with no acquired speech (although non-verbal IQ was not formally assessed). Constipation and feeding difficulties were similar to other individuals with heterozygous variants in *TLK2*. Medullary nephrocalcinosis and hydronephrosis were not observed in the previously published cohort.

**Fig. 3:**
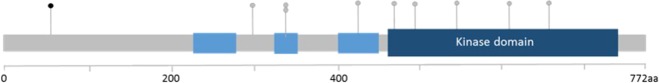
Schematic representation of the *TLK2* missense variants identified. In black, the p.(Lys55Glu) reported here; in grey, the nine variants previously reported by Reijnders et al. [3]. Annotation is according to NM_006852.3

All nine missense variants identified in the 38 families described by Reijnders et al. [3] were located either in the catalytic domain or in the coiled-coil motifs of the protein. Our variant is located at the N -terminus in a region where no functional domains are known (Fig. 3). This region, however, is expected to contain a nuclear localisation signal (NLS) as mutants lacking the first 160 amino acids fail to localise to the nucleus [6]. In addition, more than 20 phosphorylation sites, including p.Thr52 and p.Tyr70, have been identified in the N-terminal domain of TLK2, suggesting that this is potentially an important regulatory domain in vivo (https://www.phosphosite.org). In silico tools (cNLS Mapper) predict a monopartite NLS (^61^RNRKRKAEPY^70^) starting at position p.Arg61. NLSs consist of stretches of basic amino acids, primarily lysine and arginine, which bind to negative charges in the binding grooves of the transport receptor. Amino acid substitutions of lysine residues within NLSs can completely abolish their cargo nuclear import [7]. Although speculative, it may be hypothesised that the change from a positive (Lys) to a negative (Glu) side chain amino acid at the proximal position p.Lys55 may result in a change in conformation, partially occluding the binding between the NLS-cargo and the import receptor. This would lead to impaired nuclear import and in turn, diminished *TLK2* activity.

In summary, here we describe a patient with a homozygous *TLK2* variant leading to an autosomal recessive severe neurodevelopmental disorder, highlighting that certain *TLK2* variants can cause a recessive disease, as the heterozygous parents were healthy. The clinical presentation of our patient shows similarities with the previously reported patients carrying heterozygous *TLK2* variants, however the symptoms are more severe and complicated with additional features, such as cerebellar vermis hypoplasia and West syndrome. A reduction of available nuclear TLK2 would be the proposed mechanism, although this requires further functional investigation. We also highlight the importance of searching not only for de novo and dominant, but also recessive variants in *TLK2* in patients with neurodevelopmental disease. The identification of additional cases will help to further delineate the recessive *TLK2* phenotype.
